# A deep learning model to classify neoplastic state and tissue origin from transcriptomic data

**DOI:** 10.1038/s41598-022-13665-5

**Published:** 2022-06-11

**Authors:** James Hong, Laureen D. Hachem, Michael G. Fehlings

**Affiliations:** 1 Krembil Research Institute, University Health Network, 399 Bathurst Street, Suite 4W-449, Toronto, ON M5T 2S8 Canada; 2 Division of Neurosurgery, Department of Surgery, University of Toronto, 149 College Street, 5th Floor, Toronto, ON M5T 1P5 Canada; 3 Division of Neurosurgery, Krembil Neuroscience Centre, Toronto Western Hospital, University Health Network, 399 Bathurst St., Suite 4W-449, Toronto, ON M5T 2S8 Canada

**Keywords:** Computer modelling, Medical research

## Abstract

Application of deep learning methods to transcriptomic data has the potential to enhance the accuracy and efficiency of tissue classification and cell state identification. Herein, we developed a multitask deep learning model for tissue classification combining publicly available whole transcriptomic (RNA-seq) datasets of non-neoplastic, neoplastic and peri-neoplastic tissue to classify disease state, tissue origin and neoplastic subclass. RNA-seq data from a total of 10,116 patient samples processed through a common pipeline were used for model training and validation. The model achieved 99% accuracy for disease state classification (ROC-AUC of 0.98) and 97% accuracy for tissue origin (ROC-AUC of 0.99). Moreover, the model achieved an accuracy of 92% (ROC-AUC 0.95) for neoplastic subclassification. This is the first multitask deep learning algorithm developed for tissue classification employing a uniform pipeline analysis of transcriptomic data with multiple tissue classifiers. This model serves as a framework for incorporating large transcriptomic datasets across conditions to facilitate clinical diagnosis and cell-based treatment strategies.

## Introduction

Accurate and expeditious tissue classification is central to the practice of clinical medicine and biological research. Disease diagnostics, subclassification and treatment decision-making rely heavily on interpreting the identity and status of patient tissue samples^1^. Moreover, identification of cellular phenotype, stress-state and viability is critical in translating cell-based transplantation strategies to clinical practice. Methods to analyze cellular identity and tissue health have traditionally relied on a finite number of histological markers and imaging characteristics. However, advances in sequencing technology over the last decade have transformed our ability to probe tissue and expanded the availability of transcriptomic data. The cellular transcriptome provides insight into both tissue identity and response to local environmental factors, which offers a more accurate assessment of tissue status as compared to conventional histological measures^2^. While transcriptomic analyses have been employed in select diseases or cell populations^3–7^, there remains a significant need to integrate the numerous sequencing datasets available in order to probe multiple features of a tissue sample including disease state, origin, and subclass. Artificial intelligence strategies offer a promising approach to address this need and to integrate this valuable resource into clinical and research practice.

Deep learning approaches have been increasingly incorporated into clinical medicine. Unlike standard machine learning, deep learning offers the ability to train on multiple layers of neural nets, therefore affording greater flexibility and the generation of more accurate models that allow for the identification of complex patterns and granular subtyping^8^. To date, applications of deep learning methods have primarily been employed within specific disease types or in the processing of histological or radiographic images^3,9^. Recently, deep learning methods have begun to be applied to transcriptomic sequencing data in order to better understand heterogeneity in tissue samples^10–13^. Previous attempts to develop a comprehensive model based on transcriptomic data have often used standard machine learning approaches rather than multilayer neural networks^10^, and the use of non-uniform pipeline analyses of transcriptomic data processing thus lead to significant confounds in model training and output^14^. Furthermore, many previous models have been restricted to a single classifier of tissue identity^10,13^, and therefore do not capture the spectrum of disease states. Specifically, models do not distinguish non-neoplastic tissue from peri-neoplastic tissue despite there being significant differences in gene expression and microenvironment between these sample types^15^. As such, the development of an accurate and efficient transcriptomic deep learning model with multiple classifiers of tissue state is necessary.

Herein, we developed a multitask deep learning model using publicly available data from the Genotype Tissue Expression (GTEx) Project^16,17^ and The Cancer Genome Atlas (TCGA) processed through a uniform analysis pipeline. Specifically, the model contains three classifiers including disease state, tissue origin and neoplastic subclass. Our model achieved high accuracy and performance metrics for all three classifiers and serves as a framework for incorporating large transcriptomic datasets across conditions to facilitate clinical diagnosis and research development.

## Methods

### RNA sequencing data

RNA sequencing data was obtained from the Genotype Tissue Expression (GTEx) Project^16,17^ and The Cancer Genome Atlas (TCGA). As batch differences between different GTEx and TCGA submissions are well-documented, we utilized a common RNA-sequencing analysis pipeline to minimize batch effects^18^. Specifically, all raw reads were imported for alignment against hg19 in STAR, with quality control done in mRIN^19^ (mRIN < − 0.11 threshold for sample exclusion), quantification in featureCounts^20^ and batch effect correction in SVAseq^21^. In total, 10,116 patient samples were used with 17,993 genes included based on commonality across datasets (Supplementary Table 1). Dimensional reduction was performed using Sklearn package StandardScaler and principal component analysis (PCA), and 2000 principal components were used for model transformation. As a benchmark, 1000 top features selected by Random Forest and all 17,993 features (no PCA) were included in a separate run of the same models.

### Deep learning model

Our deep-learning model consists of two models executed in tandem, the first is a multi-tasking model which classifies the type (non-neoplastic, neoplastic or peri-neoplastic) and tissue origin of the tissue. The subsequent subtyping model is primed to be executed only if the sample’s tissue of origin has subtyping data available.

**Figure 1:**
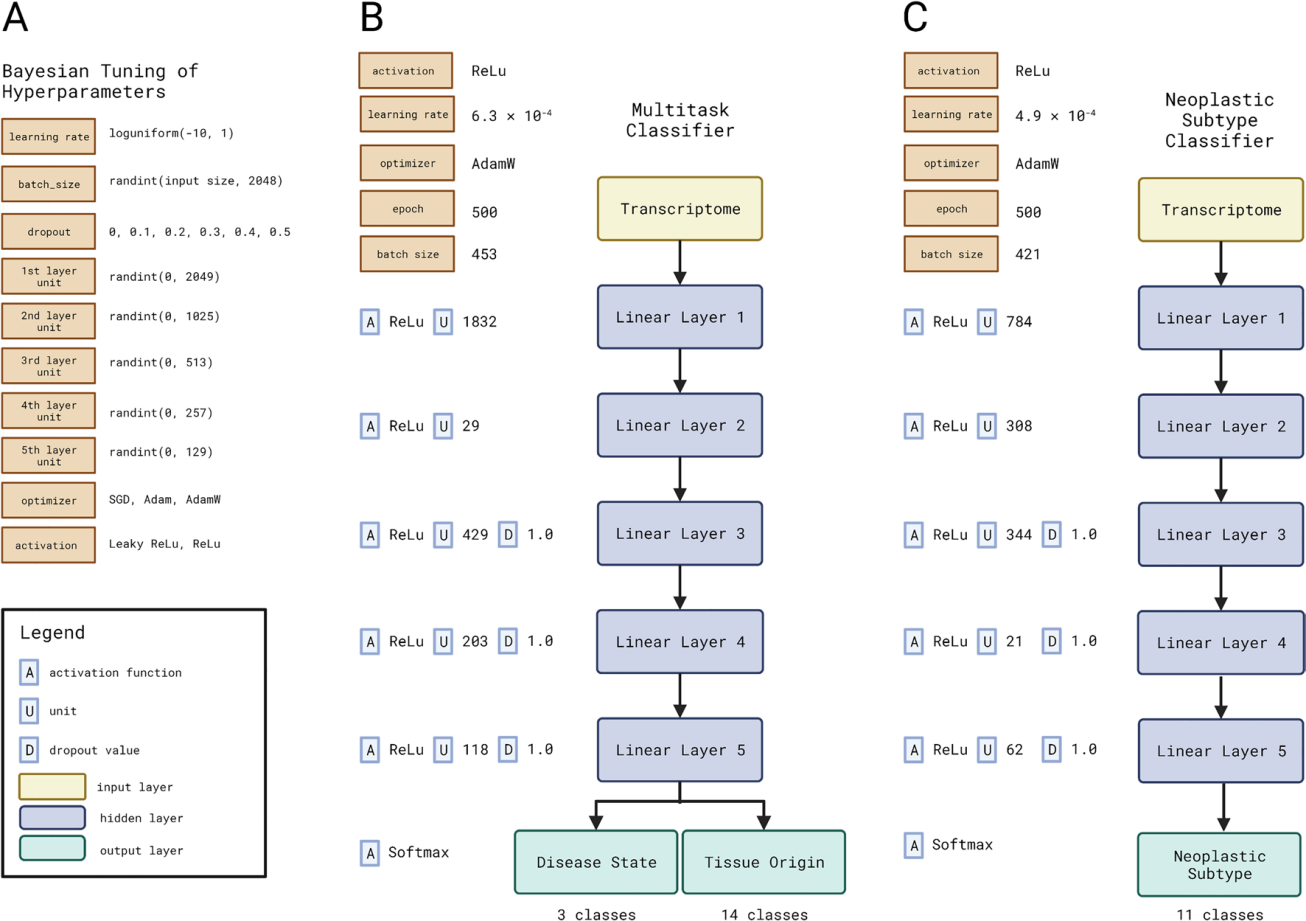
Bayesian Hyperparameter Tuning of Deep Learning Models. (**A**) Search space of hyperparameters for Bayesian tuning; (**B**) Architecture of multitask classifier for disease state and tissue origin along with tuned hyperparameters; (**C**) Architecture of neoplastic subtype classifier along with tuned hyperparameters.

Based on prior work in deep learning processing of transcriptomic data and model tuning, the encoders for both models are comprised of 7 fully connected, feed-forward neural network layers (FFNN, Fig. 1B,C). The purpose of the 5 hidden layers is to bring down the dimensionality of the input transcriptomic data. Each of these layers has a Rectified Linear Unit (ReLU) activation function on top of their outputs, which is used to restrict the output of these layers. ReLU was selected over Sigmoid or Tanh due to the lack of vanishing gradient and sparsity, ultimately resulting in faster learning and quicker convergence^22^. Hidden layers 3 through 5 also have dropout layers between their output and the next layer to reduce overfitting. In the output layer, we have task heads, which are represented by layers with a Softmax activation function. These layers map their inputs to the dimension equal to the number of classes for that task. Specifically, for the multi-tasking model, the first output head represents the type of tissue (non-neoplastic, neoplastic or normal peri-neoplastic, 3 classes) and the second output head represents the tissue origin (14 classes). Similarly, in the neoplastic subtype model, the output head presents the cancer subtype (11 classes). The Softmax activation function forces these output heads to output a probability distribution over their respective number of classes. All models were trained for 500 epochs.

### Bayesian hyperparameter tuning

We performed Bayesian hyperparameter optimization using the hyperopt package^23^, using the minimization of the cross-entropy loss as our optimization objective over 25 epochs. For each of the FFNNs, the Cartesian product of the learning rate, batch size, dropout value, unit, optimizer, and activation functions were selected as the search space (Fig. 1A). Instead of arbitrarily setting discrete values within the learning rate, batch size and units, we opted to randomize the range using the randint function. The optimal hyperparameters were then selected after 100 evaluations (Fig. 1A–C).

### Benchmarking against other Machine Learning approaches

We compared the balanced accuracy of our proposed deep learning classifiers against other machine learning algorithms in the Sckit-learn package^24^, including Decision Tree Classifier (DT), Extra Trees Classifier (ET), Support Vector Machine (SVM), Stochastic Gradient Descent (SGD) classifier, and K-nearest Neighbours Classifier (KNN). In these models, all 17,993 features were used as inputs, and a 70:15:15 ratio was used for train/validation/test splits.

## Results

### Training dataset

We used data from the Genotype Tissue Expression (GTEx) Project^16,17^ and The Cancer Genome Atlas (TCGA) for the training of our model. Specifically, 10,116 patient samples were included and processed through a uniform pipeline^18^. Seventy percent of the data was used for training with the remaining split evenly for validation (15%) and testing sets (15%). The model was trained for 500 epochs. The deep learning model consisted of a multi-tasking model that classifies disease state (non-neoplastic versus peri-neoplastic vs neoplastic) and tissue origin (14 tissue classes). A subsequent subtyping model was primed to be executed only if the sample’s tissue of origin had subtyping data available. The performance results reported here are from our proposed deep learning models with PCA dimensional reduction applied (DL PCA).

### Multi-task model: disease state and tissue origin classifiers

**Figure 2:**
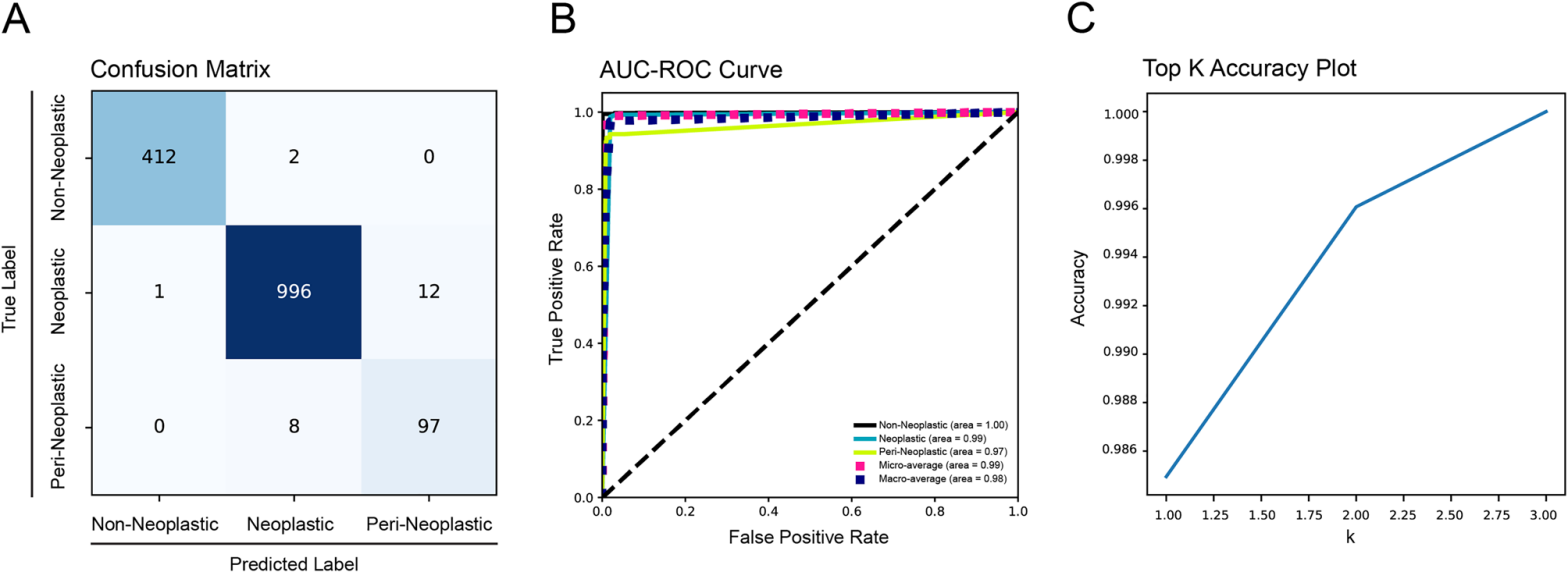
Performance of disease state classifier. (**A**) Confusion matrix of disease state classifier; (**B**) Receiver operating characteristic curve (ROC) with area under the curve (AUC); (**C**) top K accuracy plot.

The multitask portion of the model was trained on disease state (non-neoplastic versus peri-neoplastic vs neoplastic) and tissue origin (14 tissue classes). On the testing set, the disease state classifier achieved an overall accuracy of 0.99, precision of 0.99, recall of 0.99 and f1-score of 0.99 with high performance metrics for each subclass (Table 1). The associated confusion matrix (Fig. 2A) and receiver operating characteristic (ROC) curves (Fig. 2B) demonstrate that the model achieved an area under the curve (AUC) of 0.98 for disease state classification. The top K plot demonstrates that the classifier had excellent predictive accuracy without overfitting (Fig. 2C).

**Table 1:** Disease state classifier.

Accuracy	0.9882			
Balanced accuracy	0.9675			

Class	Precision	Recall	F1	Support
Non-Neoplastic	1.00	1.00	1.00	414
Neoplastic	0.99	0.99	0.99	1009
Peri-Neoplastic	0.94	0.91	0.93	105
Weighted Avg	0.99	0.99	0.99	1528

**Figure 3:**
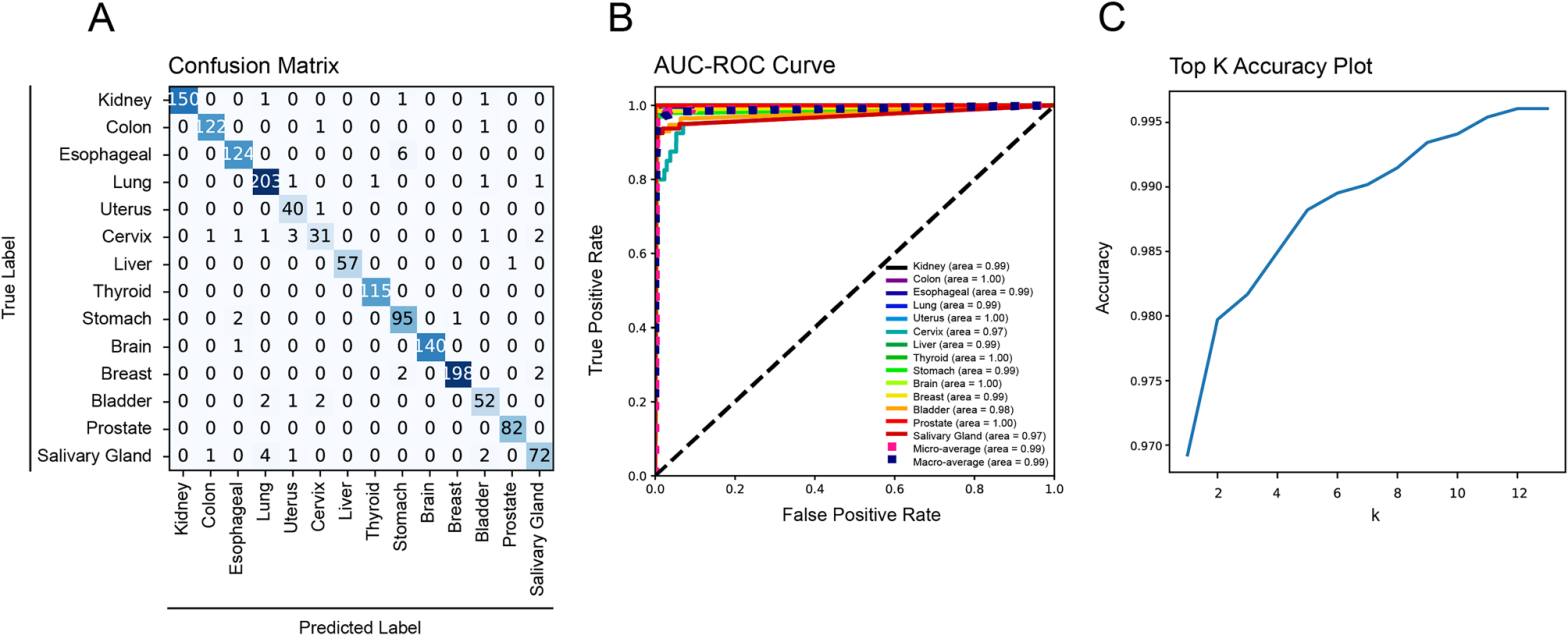
Performance of tissue origin classifier. (**A**) Confusion matrix of tissue origin classifier; (**B**) Receiver operating characteristic curve (ROC) with area under the curve (AUC); (**C**) top K accuracy plot.

In terms of the tissue origin classifier, the model achieved an accuracy of 0.97, precision of 0.97, recall of 0.97 and f1-score of 0.97 (Table 2). ROC AUCs ranged from 0.97 to 1.00 for individual tissue origins with a macro average of 0.99 (Fig. 3B) with very few misclassifications (Fig. 3A). The top K plot demonstrates that the classifier had excellent predictive accuracy without overfitting (Fig. 3C).

**Table 2:** Tissue origin classifier.

Accuracy	0.9705			
Balanced accuracy	0.9587			

Class	Precision	Recall	F1	Support
Kidney	1.00	0.99	1.00	153
Colon	0.99	1.00	1.00	124
Esophageal	0.94	0.96	0.95	130
Lung	0.95	0.98	0.96	207
Uterus	0.97	0.93	0.95	41
Cervix	0.97	0.88	0.92	40
Liver	0.98	0.98	0.98	58
Thyroid	0.99	0.99	0.99	115
Stomach	0.94	0.96	0.95	98
Brain	1.00	0.99	0.99	141
Breast	1.00	0.99	0.99	202
Bladder	0.87	0.93	0.90	57
Kidney	1.00	1.00	1.00	82
Colon	0.91	0.85	0.88	80
Weighted Avg	0.97	0.97	0.97	1528

### Neoplasm subtype classifier

**Figure 4:**
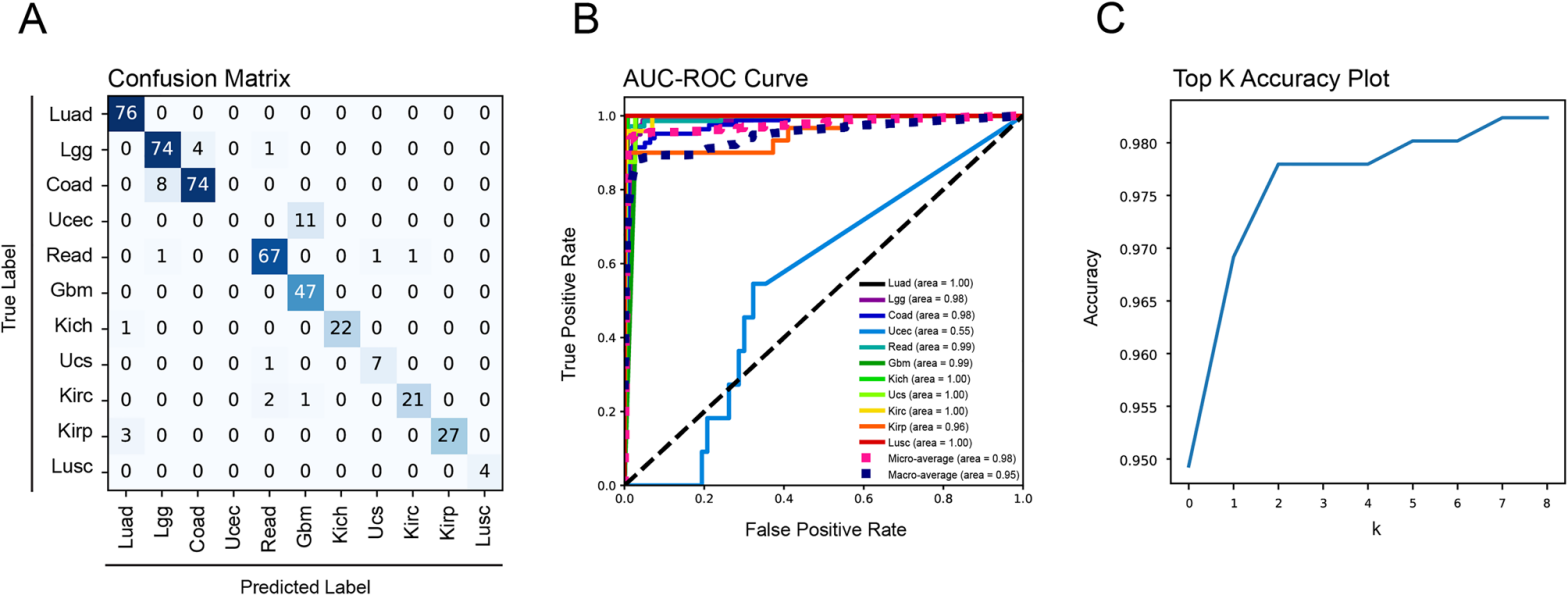
Performance of neoplastic subtype classifier. (**A**) Confusion matrix of tissue origin classifier; (**B**) Receiver operating characteristic curve (ROC) with area under the curve (AUC); (**C**) top K accuracy plot.

For tissues with multiple neoplastic subclasses (n = 11), the model was trained on an additional subtype classifier. Here, the model achieved an overall accuracy of 0.92, precision of 0.90, recall of 0.92 and F1-score of 0.91 (Table 3). ROC-AUC ranged from 0.55 to 1.00 for subtypes with a macro-average of 0.95 (Fig. 4B). It should be noted that the majority of subtypes were accurately classified, with the only exception of urterine corpus endothelial carcinoma (ucec) being classified as glioblastomas (gbm) (Fig. 4A,B). Top K plot demonstrates that the classifier had excellent predictive accuracy without overfitting (Fig. 4C).

**Table 3:** Neoplastic subtype classifier.

Accuracy	0.9229			
Balanced accuracy	0.8548			

Class	Precision	Recall	F1	Support
Luad	0.95	1.00	0.97	76
Lgg	0.89	0.94	0.91	79
Coad	0.95	0.90	0.92	82
Ucec	0.00	0.00	0.00	11
Read	0.94	0.96	0.95	70
Gbm	0.80	1.00	0.89	47
Kich	1.00	0.96	0.98	23
Ucs	0.88	0.88	0.88	8
Kirc	0.95	0.88	0.91	24
Kirp	1.00	0.90	0.95	30
Lusc	1.00	1.00	1.00	4
Weighted Avg	0.90	0.92	0.91	454

### Benchmark against various feature sets and other machine learning algorithms

**Figure 5:**
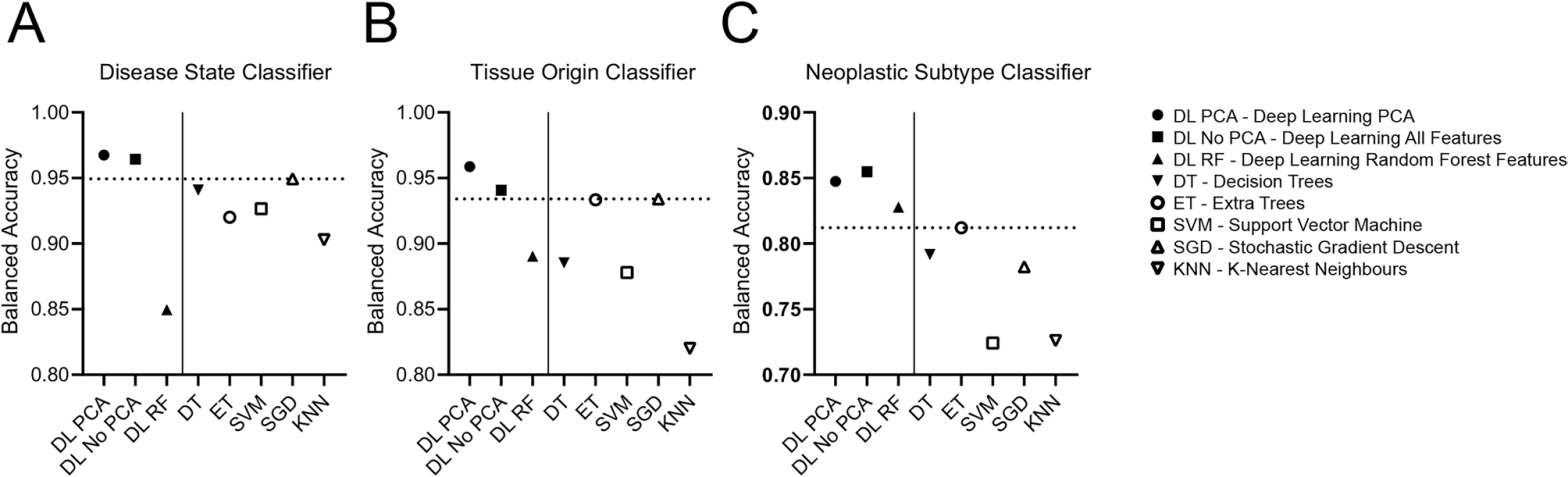
Benchmarking of deep learning classifiers and other machine learning algorithms. (**A**) Disease state classifier benchmarks; (**B**) Tissue origin classifier benchmarks; (**C**) Neoplastic subtype classifier benchmarks. Solid line separates the deep learning models from classic machine learning algorithms, and the dotted line indicates the highest balanced accuracy achieved by machine learning algorithms in each classifier.

We compared the balanced accuracy of our deep learning model (DL PCA) against deep learning models with either the full feature set (DL No PCA) or with the top 1,000 features selected by a Random Forest classifier (DL RF). Except for the neoplastic subtype classifier, DL PCA outperformed all other deep learning models. In both hyperparameter optimization and model training, DL PCA was 6 times more efficient compared to DL No PCA. The marginal gain in accuracy with the full feature set in the neoplastic subtype classifier justifies the use of PCA dimensional reduction to improve model efficiency. In all classifiers, the DL PCA model outperformed classic machine learning algorithms (DT, ET, SVM, SGD, and KNN; Fig. 5A–C, Supplementary Table 1).

## Discussion

In this study, we developed a multitask deep learning model based on the most recent compendium of RNAseq data from GTEx and TCGA. Our model achieves high performance on all metrics across the three tissue classifiers of disease state, tissue identity, and neoplastic subtype. This is the first multitask deep learning algorithm developed for tissue classification employing a uniform pipeline analysis of transcriptomic data with multiple tissue classifiers. This model serves as a foundation for incorporating large transcriptomic datasets to facilitate disease diagnosis, subclassification and treatment decision-making.

While previous models based on transcriptomic data have been attempted for neoplastic classification, these have typically employed a single neural layer and have demonstrated modest performance metrics^25,26^. The architecture of our model incorporates five feed forward layers, which affords increased accuracy and a greater number of classifiers. Indeed, our model achieves overall better accuracy with fewer samples and training epochs than previous models^14^. The ability of our model to perform well even in classes with small sample sizes is of significant value for application to rare diseases or tissue states whereby access to patient samples may be limited. Rare conditions can often pose the greatest clinical diagnostic challenges as standard histological measures may fail to achieve an accurate diagnosis^27^. Furthermore, our model was able to distinguish with high accuracy samples of non-neoplastic tissue versus peri-neoplastic tissue, thus demonstrating its utility in classifying tissue state along a spectrum of disease. The latter is defined by samples collected > 2 cm from the neoplastic margin with normal histological features and is often used as healthy controls in oncological studies^28,29^. However, normal tissue adjacent to the neoplasm has been shown to have differences in gene expression profiles compared to purely non-neoplastic tissue and as such may represent a distinct entity on the spectrum of neoplastic phenotypes^15^. To date, this distinction has not previously been incorporated into models of tissue typing.

Importantly, we used a uniform pipeline for processing RNA sequencing data from GTEx and TCGA prior to inputting into our model. Specifically, this approach employed mRIN-based exclusion of degraded samples, uniform realignment and expression quantification, along with study-specific bias correction, as previously described^18^. Previous studies have demonstrated that without uniform reprocessing and batch-correction of RNAseq data obtained from various studies, samples of different tissue identity within a single study show stronger similarities than samples of the same tissue type derived from different studies^18^. This underscores the importance of uniform processing of data prior to model training, which is a major pitfall of previous models employing a deep model architecture^14^.

Ultimately, our model provides a framework to leverage large datasets of transcriptomic data across diseases and tissue states. Tissue profiling using transcriptomic data may be of particular use in situations of diagnosing cancers of unknown primary whereby standard clinicopathologic investigations do not yield a definitive source^30^. In our model, multiple classifiers including tissue origin and subtype may offer an advantage in the clinical diagnosis of these entities. While the model was trained on a set of non-neoplastic, normal peri-neoplastic and neoplastic tissue, in the future, additional transcriptomic data can be incorporated to include classifiers of tissue stress (e.g. inflammation or oxidative stress) and cellular phenotype (e.g. specific cell lineages), thus expanding the applications of this algorithm. Tissue profiling using this approach may be a valuable tool in determining the health and viability of cell lines used for clinical applications and in comparing cellular responses to injury and disease^31^.

## Supplementary Information

Supplementary Table 1.

## Data Availability

The datasets and codes for the training and validation of the model are included in the link at: https://drive.google.com/drive/folders/1rSKDasV51ve9tbJjKtnWH9FImDFK9plb?usp=sharing. Details of the uniform pipeline analysis used for pre-processing of the RNAseq data are provided in the following link^18^: https://github.com/mskcc/RNAseqDB.
