# Supplementary material for: A deep learning model to classify neoplastic state and tissue origin from transcriptomic data
Source: Sci Rep. 2022 Jun 11;12:9669. doi: 10.1038/s41598-022-13665-5 (PMC9188604; doi:10.1038/s41598-022-13665-5)
Supplement: Supplementary file 1 — Supplementary Table 1. [file 41598_2022_13665_MOESM1_ESM.pdf]

Supplementary Table 1. Performance results of deep learning and classical machine learning algorithms.

NEOPLASTIC SUBTYPE CLASSIFIER

**Neoplastic Subtype Classifier No PCA**				
Accuracy	0.9405			
Balanced Accuracy	0.8473			
	Precision	Recall	F1	Support
0	0.98	0.98	0.98	87
1	0.00	0.00	0.00	7
2	0.93	1.00	0.96	26
3	0.98	0.93	0.95	85
4	0.96	0.97	0.97	73
5	0.93	0.96	0.95	73
6	0.88	0.90	0.89	31
7	0.92	0.92	0.92	25
8	0.82	1.00	0.90	32
9	1.00	0.91	0.95	11
10	1.00	0.75	0.86	4
Accuracy			0.94	454
Macro avg	0.85	0.85	0.85	454
Weighted Avg	0.93	0.94	0.93	454

**Neoplastic Subtype Classifier - Random Forest Features**				
Accuracy	0.9361			
Balanced Accuracy	0.8277			
	Precision	Recall	F1	Support
0	0.98	1.00	0.99	92
1	0.00	0.00	0.00	14
2	1.00	0.95	0.97	20
3	0.99	0.94	0.96	79
4	0.97	0.99	0.98	74
5	0.91	0.98	0.94	59
6	0.95	0.97	0.96	39
7	1.00	0.93	0.96	28
8	0.70	1.00	0.82	32
9	1.00	0.85	0.92	13
10	0.50	0.50	0.50	4
Accuracy			0.94	454
Macro avg	0.82	0.83	0.82	454
Weighted Avg	0.92	0.94	0.92	454

MULTITASK

Multitask No PCA – Disease State Model				
Accuracy	0.9856			
Balanced Accuracy	0.9643			
	Precision	Recall	F1	Support
0	0.98	1.00	0.99	381
1	1.00	0.99	0.99	1033
2	0.93	0.90	0.92	114
Accuracy			0.99	1528
Macro avg	0.97	0.96	0.97	1528
Weighted Avg	0.99	0.99	0.99	1528

Multitask No PCA – Tissue Origin Model				
Accuracy	0.9535			
Balanced Accuracy	0.9407			
	Precision	Recall	F1	Support
0	1.00	0.99	0.99	149
1	0.99	0.98	1.00	111
2	0.97	0.83	0.86	129
3	0.96	0.98	0.97	220
4	0.82	0.89	0.89	38
5	0.90	0.00	0.80	48
6	1.00	1.00	1.00	78
7	1.00	0.53	0.99	108
8	0.80	0.89	0.88	85
9	1.00	0.99	1.00	128
10	0.99	0.98	0.99	187
11	0.84	0.79	0.85	65
12	1.00	0.99	1.00	98
13	0.85	0.86	0.88	84
Accuracy			0.95	1528
Macro avg	0.94	0.94	0.94	1528
Weighted Avg	0.96	0.95	0.95	1528

Multitask Random Forest Features – Disease State Model				
Accuracy	0.9470			
Balanced Accuracy	0.8495			
	Precision	Recall	F1	Support
0	0.92	0.96	0.94	414
1	0.96	0.98	0.97	1009
2	0.85	0.61	0.71	105
Accuracy			0.95	1528
Macro avg	0.91	0.85	0.87	1528
Weighted Avg	0.95	0.95	0.94	1528

Multitask Random Forest Features – Tissue Origin Model				
Accuracy	0.9391			
Balanced Accuracy	0.8903			
	Precision	Recall	F1	Support
0	0.99	0.99	0.99	153
1	0.91	1.00	0.95	124
2	0.96	0.86	0.91	130
3	0.97	0.97	0.97	207
4	0.80	1.00	0.89	41
5	0.00	0.00	0.00	40
6	1.00	1.00	1.00	58
7	0.99	1.00	1.00	115
8	0.86	0.92	0.89	98
9	1.00	0.99	1.00	141
10	0.98	1.00	0.99	202
11	0.80	0.82	0.81	57
12	1.00	0.99	0.99	82
13	0.75	0.93	0.83	80
Accuracy			0.94	1528
Macro avg	0.86	0.89	0.87	1528
Weighted Avg	0.92	0.94	0.93	1528

MACHINE LEARNING

Decision Tree - Neoplastic Subtype Classifier				
Accuracy	0.8612			
Balanced Accuracy	0.7918			
	Precision	Recall	F1	Support
0	0.95	0.97	0.96	63
1	0.30	0.50	0.37	12
2	0.90	0.92	0.91	38
3	0.94	0.85	0.89	34
4	0.87	0.92	0.90	52
5	0.91	0.90	0.90	86
6	0.92	0.90	0.91	73
7	0.89	0.57	0.70	14
8	0.78	0.82	0.80	22
9	0.79	0.73	0.76	52
10	0.62	0.62	0.62	8
Accuracy			0.86	454
Macro avg	0.81	0.79	0.79	454
Weighted Avg	0.87	0.86	0.86	454

Random Forest - Neoplastic Subtype Classifier				
Accuracy	0.9273			
Balanced Accuracy	0.8147			
	Precision	Recall	F1	Support
0	0.95	1.00	0.98	63
1	1.00	0.08	0.15	12
2	0.88	0.95	0.91	38
3	1.00	0.91	0.95	34
4	0.89	0.94	0.92	52
5	0.97	0.98	0.97	86
6	1.00	0.96	0.98	73
7	1.00	0.86	0.92	14
8	0.87	0.91	0.89	22
9	0.83	1.00	0.90	52
10	0.60	0.38	0.46	8
Accuracy			0.93	454
Macro avg	0.91	0.81	0.82	454
Weighted Avg	0.93	0.93	0.92	454

ExtraTrees - Neoplastic Subtype Classifier	
Accuracy	0.9163
Balanced Accuracy	0.8121

	Precision	Recall	F1	Support
0	0.93	1.00	0.96	63
1	1.00	0.08	0.15	12
2	0.90	0.92	0.91	38
3	1.00	0.85	0.92	34
4	0.88	0.94	0.91	52
5	0.94	0.95	0.95	86
6	1.00	0.96	0.98	73
7	0.92	0.86	0.89	14
8	0.83	0.86	0.84	22
9	0.83	1.00	0.90	52
10	0.80	0.50	0.62	8
Accuracy			0.92	454
Macro avg	0.91	0.81	0.82	454
Weighted Avg	0.92	0.92	0.91	454

**SVM - Neoplastic Subtype Classifier**				
Accuracy	0.8678			
Balanced Accuracy	0.7242			
	Precision	Recall	F1	Support
0	0.84	0.98	0.91	63
1	0.00	0.00	0.00	12
2	0.92	0.95	0.94	38
3	0.95	0.59	0.73	34
4	0.92	0.87	0.89	52
5	0.82	0.98	0.89	86
6	0.99	0.93	0.96	73
7	1.00	0.64	0.78	14
8	0.72	0.82	0.77	22
9	0.79	0.96	0.87	52
10	0.67	0.25	0.36	8
Accuracy			0.87	454
Macro avg	0.78	0.72	0.74	454
Weighted Avg	0.85	0.87	0.85	454

**SGD - Neoplastic Subtype Classifier**				
Accuracy	0.8634			
Balanced Accuracy	0.7823			
	Precision	Recall	F1	Support
0	0.90	1.00	0.95	63
1	0.33	0.67	0.44	12

2	0.97	0.76	0.85	38
3	0.96	0.79	0.87	34
4	0.82	0.96	0.88	52
5	0.95	0.93	0.94	86
6	0.84	0.97	0.90	73
7	0.88	0.50	0.64	14
8	0.90	0.86	0.88	22
9	0.89	0.65	0.76	52
10	0.80	0.50	0.62	8
Accuracy			0.86	454
Macro avg	0.84	0.78	0.79	454
Weighted Avg	0.88	0.86	0.86	454

**KNN - Neoplastic Subtype Classifier**				
Accuracy	0.8370			
Balanced Accuracy	0.7261			
	Precision	Recall	F1	Support
0	0.85	0.98	0.91	63
1	0.00	0.00	0.00	12
2	0.88	0.92	0.90	38
3	0.92	0.65	0.76	34
4	0.85	0.90	0.88	52
5	0.89	0.88	0.89	86
6	0.93	0.92	0.92	73
7	1.00	0.79	0.88	14
8	0.70	0.64	0.67	22
9	0.69	0.81	0.74	52
10	0.80	0.50	0.62	8
Accuracy			0.84	454
Macro avg	0.77	0.73	0.74	454
Weighted Avg	0.83	0.84	0.83	454

**Decision Tree – Disease State Model**				
Accuracy	0.9712			
Balanced Accuracy	0.9408			
	Precision	Recall	F1	Support
0	0.98	0.99	0.98	388

1	0.98	0.98	0.98	1034
2	0.83	0.86	0.85	106
Accuracy			0.97	1528
Macro avg	0.93	0.94	0.94	1528
Weighted Avg	0.97	0.97	0.97	1528

**Decision Tree – Tissue Origin Model**				
Accuracy	0.9195			
Balanced Accuracy	0.8855			
	Precision	Recall	F1	Support
0	0.97	0.97	0.97	145
1	0.97	0.97	0.97	114
2	0.91	0.83	0.87	127
3	0.95	0.97	0.96	212
4	0.78	0.46	0.58	46
5	0.56	0.80	0.65	44
6	0.93	0.97	0.95	72
7	1.00	0.98	0.99	110
8	0.76	0.85	0.80	80
9	1.00	0.99	1.00	131
10	0.95	0.94	0.95	204
11	0.81	0.83	0.82	63
12	0.98	0.98	0.98	95
13	0.87	0.86	0.86	85
Accuracy			0.92	1528
Macro avg	0.89	0.89	0.88	1528
Weighted Avg	0.92	0.92	0.92	1528

Random Forest – Disease State Model				
Accuracy	0.9849			
Balanced Accuracy	0.9497			
	Precision	Recall	F1	Support
0	0.99	1.00	0.99	388
1	0.99	0.99	0.99	1034
2	0.95	0.86	0.90	106
Accuracy			0.98	1528
Macro avg	0.97	0.95	0.96	1528
Weighted Avg	0.98	0.98	0.98	1528

Random Forest – Tissue Origin Model				
Accuracy	0.9718			
Balanced Accuracy	0.9604			
	Precision	Recall	F1	Support
0	0.99	0.99	0.99	145
1	1.00	1.00	1.00	114
2	0.97	0.84	0.90	127
3	0.98	1.00	0.99	212
4	0.93	0.93	0.93	46
5	0.95	0.82	0.88	44
6	1.00	1.00	1.00	72
7	1.00	1.00	1.00	110
8	0.82	1.00	0.90	80
9	1.00	0.99	1.00	131
10	0.99	1.00	1.00	204
11	0.98	0.90	0.94	63
12	0.99	1.00	0.99	95
13	0.91	0.96	0.94	85
Accuracy			0.97	1528
Macro avg	0.97	0.96	0.96	1528
Weighted Avg	0.97	0.97	0.97	1528

Extra Trees – Disease State Model				
Accuracy	0.9784			
Balanced Accuracy	0.9200			
	Precision	Recall	F1	Support
0	0.99	0.99	0.99	388
1	0.98	0.99	0.99	1034
2	0.94	0.77	0.85	106
Accuracy			0.98	1528
Macro avg	0.97	0.92	0.94	1528
Weighted Avg	0.98	0.98	0.98	1528

Extra Trees – Tissue Origin Model				
Accuracy	0.9555			
Balanced Accuracy	0.9334			
	Precision	Recall	F1	Support
0	0.99	0.99	0.99	145
1	0.99	1.00	1.00	114
2	0.96	0.84	0.90	127
3	0.88	0.99	0.93	212
4	0.93	0.89	0.91	46
5	1.00	0.73	0.84	44
6	1.00	0.99	0.99	72
7	1.00	0.99	1.00	110
8	0.84	0.96	0.90	80
9	1.00	0.99	1.00	131
10	1.00	1.00	1.00	204
11	0.98	0.78	0.87	63
12	0.99	1.00	0.99	95
13	0.85	0.92	0.88	85
Accuracy			0.96	1528
Macro avg	0.96	0.93	0.94	1528
Weighted Avg	0.96	0.96	0.95	1528

SVM – Disease State Model				
Accuracy	0.9791			
Balanced Accuracy	0.9265			
	Precision	Recall	F1	Support
0	0.98	0.99	0.99	388
1	0.98	0.99	0.99	1034
2	0.94	0.79	0.86	106
Accuracy			0.98	1528
Macro avg	0.97	0.93	0.94	1528
Weighted Avg	0.98	0.98	0.98	1528

SVM– Tissue Origin Model				
Accuracy	0.9162			
Balanced Accuracy	0.8780			
	Precision	Recall	F1	Support
0	0.99	0.98	0.98	145
1	0.98	0.96	0.97	114
2	0.83	0.83	0.83	127
3	0.84	0.98	0.91	212
4	0.80	0.80	0.80	46
5	0.83	0.55	0.66	44
6	1.00	0.97	0.99	72
7	1.00	0.99	1.00	110
8	0.76	0.86	0.81	80
9	1.00	0.98	0.99	131
10	0.95	0.98	0.97	204
11	0.96	0.68	0.80	63
12	1.00	0.93	0.96	95
13	0.81	0.81	0.81	85
Accuracy			0.92	1528
Macro avg	0.91	0.88	0.89	1528
Weighted Avg	0.92	0.92	0.91	1528

SGD – Disease State Model				
Accuracy	0.9830			
Balanced Accuracy	0.9493			
	Precision	Recall	F1	Support
0	0.98	1.00	0.99	388
1	0.99	0.99	0.99	1034
2	0.92	0.86	0.89	106
Accuracy			0.98	1528
Macro avg	0.96	0.95	0.96	1528
Weighted Avg	0.98	0.98	0.98	1528

SGD – Tissue Origin Model				
Accuracy	0.9535			
Balanced Accuracy	0.9341			
	Precision	Recall	F1	Support
0	0.99	1.00	0.99	145
1	0.97	0.99	0.98	114
2	0.94	0.92	0.93	127
3	0.95	0.92	0.94	212
4	0.89	0.89	0.89	46
5	0.76	0.66	0.71	44
6	0.97	1.00	0.99	72
7	1.00	1.00	1.00	110
8	0.90	0.95	0.93	80
9	1.00	1.00	1.00	131
10	0.99	0.99	0.99	204
11	0.95	0.83	0.88	63
12	0.99	1.00	0.99	95
13	0.82	0.93	0.87	85
Accuracy			0.95	1528
Macro avg	0.94	0.93	0.93	1528
Weighted Avg	0.95	0.95	0.95	1528

KNN – Disease State Model				
Accuracy	0.9561			
Balanced Accuracy	0.9029			
	Precision	Recall	F1	Support
0	0.91	0.99	0.95	388
1	0.99	0.96	0.98	1034
2	0.83	0.75	0.79	106
Accuracy			0.96	1528
Macro avg	0.91	0.90	0.91	1528
Weighted Avg	0.96	0.96	0.96	1528

KNN – Tissue Origin Model				
Accuracy	0.8665			
Balanced Accuracy	0.8200			
	Precision	Recall	F1	Support
0	0.94	0.97	0.95	145
1	0.71	0.89	0.79	114
2	0.78	0.75	0.76	127
3	0.79	0.91	0.84	212
4	0.82	0.80	0.81	46
5	0.64	0.32	0.42	44
6	1.00	0.99	0.99	72
7	0.99	1.00	1.00	110
8	0.78	0.70	0.74	80
9	1.00	1.00	1.00	131
10	0.92	0.92	0.92	204
11	0.91	0.49	0.64	63
12	0.98	0.96	0.97	95
13	0.77	0.80	0.79	85
Accuracy			0.87	1528
Macro avg	0.86	0.82	0.83	1528
Weighted Avg	0.87	0.87	0.86	1528
